# Patient and physician perceptions of seasonal allergic rhinitis and allergen immunotherapy: a parallel physician patient survey

**DOI:** 10.1186/s13223-020-0412-8

**Published:** 2020-02-21

**Authors:** Anne K. Ellis, Jean Boursiquot, Stuart Carr, François Graham, Marie-Soleil Masse

**Affiliations:** 1 Division of Allergy & Immunology, Department of Medicine, Queen’s University, Kingston, ON Canada; 2 Division of Allergy and Clinical Immunology, Centre hospitalier universitaire de Québec, Laval University, Quebec City, QC Canada; 3 Department of Pediatrics, University of Alberta, Edmonton, AB Canada; 4 Division of Allergy and Immunology, Department of Medicine, Université de Montréal, Quebec, Canada

**Keywords:** Allergen immunotherapy, Patient preference, Seasonal allergies, Allergist preference, Hay fever, Allergic rhinitis, Sublingual immunotherapy, Subcutaneous immunotherapy

## Abstract

**Background:**

The Allergy Patient Identification for Immunotherapy (AsPIRe) program was a parallel physician and patient survey. The objectives were to examine physician and patient perceptions of seasonal allergy symptoms and their impact on patients, and to examine patient and physician attitudes to allergen immunotherapy (AIT) for seasonal allergies. AsPIRe was led by a steering committee and received research ethics board clearance from Queen’s University.

**Methods:**

Allergists (17) from across Canada enrolled in the AsPIRe program and completed an on-line survey to collect demographic information and baseline perceptions. Allergists then recruited patients and completed paper-based parallel physician and patient questionnaires. Patients received an AIT informational booklet with their questionnaire. Patients who were AIT-naïve with no contraindication to AIT and 12 years of age and older met the inclusion criteria.

**Results:**

The survey was in field from February 2018 to June 2018. A total of 141 allergist surveys and 136 patient surveys were completed. Mean age of patients was 30 years old (range 12–70). Fifty-seven percent of patients reported prior knowledge of AIT. Seventy-two percent of patients reported seasonal allergies of longer than 5 years duration and in this subset of patients, 46% were at their first allergist visit. Seventy-three percent of all patients indicated they would be likely or very likely to try sublingual immunotherapy (SLIT), if recommended by their allergist compared to 36% for subcutaneous immunotherapy (SCIT). Conversely, 10% of patients reported they would be unlikely or very unlikely to try SLIT compared to 46% of patients who would be unlikely or very unlikely to try SCIT if recommended by their allergist.

**Conclusions:**

In this particular study cohort, there was a gap in perception between allergists and their patients as to the impact of allergy symptoms on daily life. Patients reported being more frequently impacted vs. their physician’s assessment. When asked about preference for AIT options, Canadian patients reported they were more likely to follow their allergists’ recommendation for initiation of SLIT compared to SCIT.

## Introduction

The number of Canadians affected by allergies is estimated at 8.4 million [1]. The prevalence of physician-diagnosed allergic rhinitis was estimated at 20% in a structured telephone interview of 3671 Canadians over the age of 18 [5].

Allergic rhinitis (AR) symptoms present a substantial burden to patients’ everyday lives. When comparing the burden of allergic rhinitis to depression, hypertension and diabetes mellitus, allergic rhinitis was second only to depression in its impact on work productivity and restriction of daily activities [2]. An online survey examined the impact of nasal and ocular allergy symptoms in 1001 patients [3]. Fifty percent of patients reported impaired daily activities and/or increased distraction, irritability, fatigue and frustration as a result of symptoms [3]. Allergic rhinitis symptoms have also been found to negatively impact both sexual function and sleep as evaluated by the Rhinosinusitis Disability Index, a validated outcomes tool [4].

Despite treatment, a high percentage of AR patients have uncontrolled symptoms. In a structured telephone interview of Canadian patients with physician diagnosed allergic rhinitis, 66% indicated that their lifestyle was limited despite using medications for their allergies and 61% felt their symptoms were only somewhat or poorly/not controlled [5].

Allergen specific immunotherapy (AIT) is the only allergy treatment that modifies the underlying disease process [6]. Two forms of AIT are approved for use in Canada, subcutaneous immunotherapy (SCIT) delivered via injection, or sublingual immunotherapy (SLIT) delivered in a tablet form.

The Allergy Patient Identification for Immunotherapy (AsPIRe) Program investigated similarities and differences between allergist and patient perceptions of seasonal allergy symptoms and their impact on daily living. The program also looked at patient and physician attitudes toward available allergen immunotherapy (AIT) modalities and patients’ likely acceptance of AIT if offered as part of a seasonal allergy treatment approach.

## Methods

Thirty-nine allergists from across Canada were invited to participate in the AsPIRe Program. Twenty-nine enrolled and completed an on-line survey to collect demographic information and baseline perceptions. Nineteen allergists recruited patients who met the inclusion criteria: AIT-naïve seasonal allergy patients with no contraindication to AIT and at least 12 years of age.

For each patient who was selected to participate and met the inclusion criteria, the allergist and patient completed paper-based parallel allergist and patient questionnaires. Patients also received an AIT informational booklet with their questionnaire. The allergist survey was 7 questions in length and the patient survey consisted of 12 questions (surveys available as Additional files 1, 2).

Results were tabulated and where comparable questions were posed to both the patient and physician, cross-tabulations of the results were done to examine any similarities or patterns in the responses. A p-value test was done to evaluate diagonal symmetry between the patient and physician distribution of responses. A p-value that is greater than 0.05 means there is no evidence for lack of symmetry.

Following tabulation of the parallel allergist and patient paper-based surveys, the results were communicated to the participating allergists.

## Results

### Baseline characteristics

The survey was in field from February 2018 to June 2018. A total of 141 allergist surveys and 136 patient surveys were completed. Provincial breakdown of surveys returned was 40% from Ontario, Alberta 24%, Quebec 24% and British Columbia 12%. The mean age of patients was 30 years old (range 12–70), and 44% were male, 56% female. 72% of patients reported seasonal allergies of longer than 5 years duration and in this subset of patients, 46% were at their first allergist visit.

### Impact of allergy symptoms

When questioned about the impact of specific symptoms, both patients and allergists rated nasal symptoms as the most bothersome. On a five-point scale, the mean was calculated and patients reported blocked nose (3.9) and sneezing (3.7) as the most bothersome while allergists rated blocked nose (3.7) and runny nose (3.4) as the most bothersome. Conversely, headaches and quantity and quality of sleep were rated least bothersome by both patients and allergists. Agreement between the patient and allergist was highest for blocked nose being the most bothersome symptom (p-value test of symmetry p = 0.66).

**Fig. 1:**
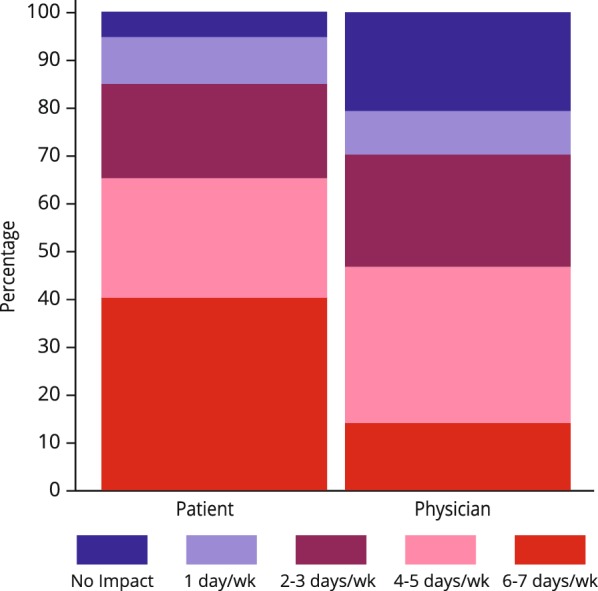
Physician and patient response: effect of symptoms on daily life (daily living activities/regular day-to-day life)

Analysis of the data showed that while 40% of patients reported being affected by their allergy symptoms 6–7 days a week, only 14% of allergists felt their patients suffered with a similar frequency (Fig. 1). Both allergists and patients reported attendance and performance at work and school as the quality of life factors the least impacted on a daily basis by their allergy symptoms.

### Awareness of AIT

**Fig. 2:**
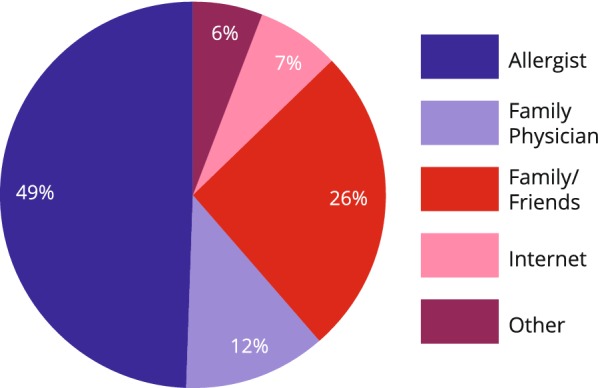
Patient awareness of AIT: source of information

Fifty-eight percent of patients reported they were aware of AIT before completing the survey. Of the 58% who were aware of AIT, 49% had been informed by their allergist while 26% had heard about AIT from friends and family (Fig. 2). Of note, only 7% of patients had been made aware of AIT by the internet.

### Patient preference for type of AIT

**Fig. 3:**
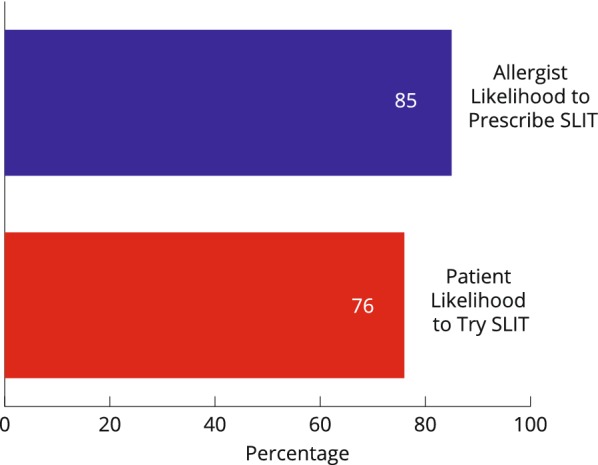
Likelihood to use SLIT: allergist likelihood to prescribe versus patient likelihood to use

**Fig. 4:**
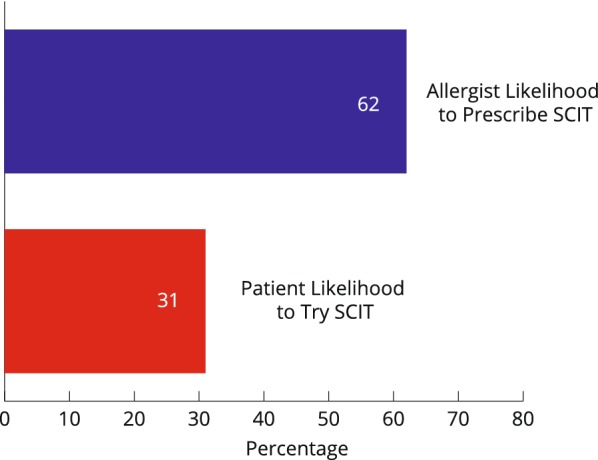
Likelihood to use SCIT: allergist likelihood to prescribe versus patient likelihood to use

Patient responses showed that 76% of patients indicated they would be likely or very likely to try sublingual immunotherapy (SLIT) if recommended by their allergist compared to 31% for subcutaneous immunotherapy (SCIT) (Figs. 3 and 4).

Conversely, 8% of patients reported they would be unlikely or very unlikely to try SLIT compared to 50% of patients who would be unlikely or very unlikely to try SCIT if recommended by their allergist.

## Discussion

Comparing the patient and physician questionnaires demonstrated a gap in perception between allergists and their patients as to the impact of allergy symptoms on daily life. In this cohort, compared to their allergists, patients were nearly 3 times more likely to report that allergy symptoms impacted their daily life 6–7 days a week. The magnitude of difference between the impact on daily life assessed by allergists and the impact reported by patients would be expected to be of clinical significance. This underscores that patients are not as well controlled as their allergists may conclude.

To provide patients with better allergic rhinitis control, their management requires collaboration between allergists and patients, including a presentation of all therapeutic options. The Canadian Society of Allergy and Clinical Immunology recommends immunotherapy should be considered in patients whose rhinitis and lower airway symptoms are triggered by allergen exposure and who have not achieved sufficient control with or have not tolerated conventional pharmacotherapy, or do not want to be on ongoing or long-term pharmacotherapy [6].

Patients showed a c preference for initiating SLIT vs SCIT if it were to be recommended by their allergist. Half of patients were not likely or not at all likely to initiate SCIT if it were to be recommended by their allergist. In contrast, only 8% of patients were not likely or not at all likely to follow their allergist’s recommendation to initiate with SLIT. These results support the patient preference for SLIT inferred by Damm et al. [7] from their discrete choice study showing preferences for fewer physician visits and visits of shorter duration as their preferred attributes of an AIT.

There are clear limitations to this study. Firstly, we had a small sample size and potential selection bias. Allergists who were approached to participate were known to be more generally receptive to participating in studies and to prescribing AIT. Geographical representation was not uniform across the country as surveys were only completed by allergists and patients in the provinces of British Columbia, Alberta, Ontario and Quebec. A further limitation was the non-randomized patient selection. Participating allergists decided which individual patients to approach for participation and this could have introduced selection bias. Additionally, while the questionnaires used in the parallel physician–patient survey were developed by the steering committee members based on their clinical experience, and available in English and French based on patient preference, they were not validated tools.

## Conclusions

In this particular study cohort, there was a gap in perception between allergists and their patients as to the impact of allergy symptoms on daily life. Patients reported being more frequently impacted versus their physician’s assessment. When asked about preference for AIT options, this cohort of Canadian patients reported they were more likely to follow their allergists’ recommendation for initiation of SLIT compared to SCIT.

## Supplementary information

**Additional file 1.** AsPIRe program patient survey.

**Additional file 2.** AsPIRe program physician survey.

## Data Availability

The datasets used and analysed during the current study are available from the corresponding author on reasonable request.
