# Supplementary material for: Patient and physician perceptions of seasonal allergic rhinitis and allergen immunotherapy: a parallel physician patient survey
Source: Allergy Asthma Clin Immunol. 2020 Feb 21;16:15. doi: 10.1186/s13223-020-0412-8 (PMC7035743; doi:10.1186/s13223-020-0412-8)
Supplement: Supplementary file 1 — Additional file 1. AsPIRe program patient survey. [file 13223_2020_412_MOESM1_ESM.docx]

**Patient Survey**

.

1. How old are you?
2. Please indicate your gender:
   - Female
   - Male
   - Other ______________
3. How long have you had seasonal allergy symptoms?
   - Less than a year
   - Between 1 and 5 years
   - Between 6 and 10 years
   - More than 10 years
4. How often do you see an allergist for your seasonal allergies?
   - This is my first appointment with an allergist
   - This is a regular follow-up appointment for my seasonal allergies that occurs every ____ months
   - I see my allergist when my allergies are bothering me, approximately every ____ ___
5. For this question, think about allergy season, when your symptoms are at their worst. Mark on a scale of 0 to 5 how these seasonal allergy symptoms affect you (0 = not at all bothersome, 10 = very bothersome):

- Runny nose

0-----------------------------------------------------------5--------------------------------------------------10

- Sneezing

0-----------------------------------------------------------5--------------------------------------------------10

- Blocked nose

0-----------------------------------------------------------5--------------------------------------------------10

- Nasal itch

0-----------------------------------------------------------5--------------------------------------------------10

- Watery eyes

0-----------------------------------------------------------5--------------------------------------------------10

- Red or itchy eyes

0-----------------------------------------------------------5--------------------------------------------------10

- Itchy throat or palate

0-----------------------------------------------------------5--------------------------------------------------10

- Headaches

0-----------------------------------------------------------5--------------------------------------------------10

- How long you sleep

0-----------------------------------------------------------5--------------------------------------------------10

- How well you sleep

0-----------------------------------------------------------5--------------------------------------------------10

1. During peak allergy season, how much do your allergies affect you? Rank using the following scale:
   - - No effect
     - 1 day per week affected
     - 2-3 days per week affected
     - 4-5 days per week affected
     - 6-7 days per week affected

Please check the box that applies for each question

| How much do your allergies affect your? | No effect | 1 day per week affected | 2-3 days per week affected | 4-5 days per week affected | 6-7 days per week affected |
| --- | --- | --- | --- | --- | --- |
| Regular day to day life? |  |  |  |  |  |
| How long you sleep? |  |  |  |  |  |
| How well you sleep? |  |  |  |  |  |
| Social activities or sports? |  |  |  |  |  |
| Performance at work or school? |  |  |  |  |  |
| Attendance at work or school? |  |  |  |  |  |

1. Overall, during peak allergy season, how much do your allergies bother you in your daily life?

- Not at all
- A little
- Somewhat
- A lot

For the next questions, please read the pamphlet on allergy immunotherapy before answering.

1. Before reading the pamphlet, had you ever heard about allergy immunotherapy?
   - Yes
   - No
2. If you answered “yes”, where did you hear about allergy immunotherapy?

- My allergist
- My family doctor
- Family member or friend
- Internet
- Other sources like TV, radio or magazine

1. Based on the information you have just read, mark on the scale whether you are “less likely or “more likely” to choose a therapy that:

A series of shots given at the doctor’s office

Less likely to choose Neutral More likely to choose

1-----------------------------------------------------------3--------------------------------------------------5

Tablets that you put under your tongue, that are taken at home

Less likely to choose Neutral More likely to choose

1-----------------------------------------------------------3--------------------------------------------------5

Requires a visit to the doctor for shots weekly to build up to the maintenance dose and then monthly maintenance shots

Less likely to choose Neutral More likely to choose

1-----------------------------------------------------------3--------------------------------------------------5

Requires visits to the doctor to get a prescription for tablets and for follow-up

Less likely to choose Neutral More likely to choose

1-----------------------------------------------------------3--------------------------------------------------5

Might cause a serious side effect like anaphylaxis

Less likely to choose Neutral More likely to choose

1-----------------------------------------------------------3--------------------------------------------------5

Might cause local side effects like swelling where you get the shot

Less likely to choose Neutral More likely to choose

1-----------------------------------------------------------3--------------------------------------------------5

Might cause local side effects like swelling or itching under the tongue

Less likely to choose Neutral More likely to choose

1-----------------------------------------------------------3--------------------------------------------------5

Whether I have a drug plan that will pay for the treatment

Less likely to choose Neutral More likely to choose

1-----------------------------------------------------------3--------------------------------------------------5

1. If your allergist recommended SLIT allergy immunotherapy (tablets taken by mouth), how likely is it that you would try it, where 0 is not at all likely and 10 is very likely?

Not at all likely Neutral Very likely

1-----------------------------------------------------------3--------------------------------------------------5

1. If your allergist recommended SCIT allergy immunotherapy (shots taken at the allergist’s office), how likely is it that you would try it, where 1 is not at all likely and 5 is very likely?

Not at all likely Neutral Very likely

1-----------------------------------------------------------3--------------------------------------------------5
