# Supplementary material for: Patient and physician perceptions of seasonal allergic rhinitis and allergen immunotherapy: a parallel physician patient survey
Source: Allergy Asthma Clin Immunol. 2020 Feb 21;16:15. doi: 10.1186/s13223-020-0412-8 (PMC7035743; doi:10.1186/s13223-020-0412-8)
Supplement: Supplementary file 2 — Additional file 2. AsPIRe program physician survey. [file 13223_2020_412_MOESM2_ESM.docx]

**Physician Survey**

Please answer the following questions based on your knowledge and observations of this patient.

1. How long has this patient had seasonal allergy symptoms?
   - Less than a year
   - Between 1 and 5 years
   - Between 6 and 10 years
   - More than 10 years
2. How often do you see this patient for your seasonal allergies?
   - This is their first appointment with an allergist
   - This is a regular follow-up appointment for their seasonal allergies, occurs every ____ months
   - I see this patient on an “as needed” basis, approximately ____ times per _____
3. Based on your discussions and clinical observation of this patient, please indicate on a scale of 0 to 10 how the following seasonal allergy symptoms at their peak affect your patient (0 = not at all bothersome, 10 = very bothersome):

Runny nose

0-----------------------------------------------------------5--------------------------------------------------10

Sneezing

0-----------------------------------------------------------5--------------------------------------------------10

Blocked nose

0-----------------------------------------------------------5--------------------------------------------------10

Nasal itch

0-----------------------------------------------------------5--------------------------------------------------10

Watery eyes

0-----------------------------------------------------------5--------------------------------------------------10

Red or itchy eyes

0-----------------------------------------------------------5--------------------------------------------------10

Itchy throat or palate

0-----------------------------------------------------------5--------------------------------------------------10

Headaches

0-----------------------------------------------------------5--------------------------------------------------10

Quantity of sleep

0-----------------------------------------------------------5--------------------------------------------------10

Quality of sleep

0-----------------------------------------------------------5--------------------------------------------------10

1. Based on your observation of this patient during peak allergy season, how much do allergy symptoms affect their daily life? Rank using the following scale:
   - 1. No impact
     2. 1 day per week affected
     3. 2-3 days per week affected
     4. 4-5 days per week affected
     5. 6-7 days per week affected

Please check the box that applies for each statement.

|  | No impact | 1 day per week affected | 2-3 days per week affected | 4-5 days per week affected | 6-7 days per week affected |
| --- | --- | --- | --- | --- | --- |
| Daily living activites |  |  |  |  |  |
| Quality of sleep |  |  |  |  |  |
| Amount of sleep |  |  |  |  |  |
| Participation in social activities |  |  |  |  |  |
| Performance at work or school |  |  |  |  |  |
| Attendance at work or school |  |  |  |  |  |

1. Overall, how bothersome are their allergies to this patient’s daily life during peak allergy season?

- Not at all
- Minimally
- Moderately
- Significantly

1. For this patient, please rate the impact of the following factors on your decision to consider/recommend immunotherapy treatment decision, where 1 is less likely to use AIT, 3 is neutral, and 5 is more likely to use AIT

Satisfactory efficacy of their current treatment for seasonal allergy

Less likely to use AIT Neutral More likely to use AIT

1-----------------------------------------------------------3--------------------------------------------------5

Suboptimal efficacy of their current treatment for seasonal allergy

Less likely to use AIT Neutral More likely to use AIT

1-----------------------------------------------------------3--------------------------------------------------5

Potential efficacy of AIT in reducing allergic response

Less likely to use AIT Neutral More likely to use AIT

1-----------------------------------------------------------3--------------------------------------------------5

Compliance or persistence with SCIT

Less likely to use AIT Neutral More likely to use AIT

1-----------------------------------------------------------3--------------------------------------------------5

Compliance or persistence with SLIT

Less likely to use AIT Neutral More likely to use AIT

1-----------------------------------------------------------3--------------------------------------------------5

Potential for systemic adverse events with SCIT

Less likely to use AIT Neutral More likely to use AIT

1-----------------------------------------------------------3--------------------------------------------------5

Potential for systemic adverse events with SLIT

Less likely to use AIT Neutral More likely to use AIT

1-----------------------------------------------------------3--------------------------------------------------5

Patient access to private or public coverage for the costs associated with SCIT

Less likely to use AIT Neutral More likely to use AIT

1-----------------------------------------------------------3--------------------------------------------------5

Patient access to private or public coverage for the costs associated with SLIT

Less likely to use AIT Neutral More likely to use AIT

1-----------------------------------------------------------3--------------------------------------------------5

1. Based on your knowledge of this patient, how do you rate the following factors in your choice of treatment, where 1 is “less likely to prescribe” and 5 is “more likely to prescribe”:

Injections given at the doctor’s office

Less likely to prescribe Neutral More likely to prescribe

1-----------------------------------------------------------3--------------------------------------------------5

Sublingual tablets taken at home

Less likely to prescribe Neutral More likely to prescribe

1-----------------------------------------------------------3--------------------------------------------------5

Required number of visits to the doctor for injections

Less likely to prescribe Neutral More likely to prescribe

1-----------------------------------------------------------3--------------------------------------------------5

Required number of visits to the doctor for sublingual tablets and follow-up

Less likely to prescribe Neutral More likely to prescribe

1-----------------------------------------------------------3--------------------------------------------------5

Potential for a serious side effect like anaphylaxis

Less likely to prescribe Neutral More likely to prescribe

1-----------------------------------------------------------3--------------------------------------------------5

Potential for local side effects at the site of injection

Less likely to prescribe Neutral More likely to prescribe

1-----------------------------------------------------------3--------------------------------------------------5

Potential for local side effects under the tongue

Less likely to prescribe Neutral More likely to prescribe

1-----------------------------------------------------------3--------------------------------------------------5

Patient preference for SCIT

Less likely to prescribe Neutral More likely to prescribe

1-----------------------------------------------------------3--------------------------------------------------5

Patient preference for SLIT

Less likely to prescribe Neutral More likely to prescribe

1-----------------------------------------------------------3--------------------------------------------------5

Cost to the patient

Less likely to prescribe Neutral More likely to prescribe

1-----------------------------------------------------------3--------------------------------------------------5
